# In vivo regulation of the monocyte phenotype by *Mycobacterium marinum* and the ESX-1 type VII secretion system

**DOI:** 10.1038/s41598-025-88212-z

**Published:** 2025-02-07

**Authors:** Kristina Munke, Line Wulff, Julia Lienard, Fredric Carlsson, William W. Agace

**Affiliations:** 1 Department of Experimental Medical Science, Lund University, Lund, Sweden; 2 Department of Biology, Lund University, Lund, Sweden; 3 Department of Immunology and Microbiology, LEO Foundation Skin Immunology Research Centre, University of Copenhagen, Copenhagen, Denmark; 4 Department of Physiology and Pharmacology, University of Calgary, Calgary, AB Canada

**Keywords:** Immunology, Microbiology

## Abstract

Pathogenic mycobacteria require the conserved ESX-1 type VII secretion system to cause disease. In a murine *Mycobacterium marinum* infection model we previously demonstrated that infiltrating monocytes and neutrophils represent the major bacteria-harbouring cell populations in infected tissue. In the current study we use this model, in combination with scRNA sequencing, to assess the impact of *M. marinum* infection on the transcriptional profile of infiltrating Ly6C⁺MHCII⁺ monocytes in vivo. Our findings demonstrate that infection of infiltrating monocytes with *M. marinum* alters their cytokine expression profile, induces glycolytic metabolism, hypoxia-mediated signaling, nitric oxide synthesis, tissue remodeling, and suppresses responsiveness to IFNγ. We further show that the transcriptional response of bystander monocytes is influenced by ESX-1-dependent mechanisms, including a reduced responsiveness to IFNγ. These findings suggest that mycobacterial infection has pleiotropic effects on monocyte phenotype, with potential implications in bacterial growth restriction and granuloma formation.

## Introduction

Tuberculosis, caused by the intracellular pathogen *Mycobacterium tuberculosis*, remains one of the top infectious killers worldwide and about one-fourth of the global population is estimated to be latently infected with the bacteria^1^, although the high prevalence of latency is under debate^2^. During the initial stages of *M. tuberculosis* infection, inhaled bacteria are phagocytosed by alveolar macrophages that migrate into the lung interstitium^3^. This process triggers the recruitment of blood monocytes, which differentiate in situ into various myeloid populations and acquire bacteria within the tissue^3–5^. Studies attempting to delineate the role of alveolar macrophages and interstitial macrophages during *M. tuberculosis* infection indicate that the former represent a nutritionally permissive environment for bacterial growth, while the latter a more stressful and restrictive environment^6^. The significance of monocyte derived cells in the immune response to pathogenic mycobacteria is highlighted by findings in CCR2-deficient mice, where impaired monocyte recruitment leads to exaggerated disease progression and delayed T-cell activation^7–11^.

The ESX-1 type VII secretion system enables transport of bacterial proteins across the mycobacterial cell wall and is a major virulence determinant during mycobacterial infection^12^. ESX-1’s structural and secreted effector proteins are primarily encoded by genes located within and adjacent to the genomic “region of difference 1” (RD1)^12^, defined by the corresponding deletion in the *Mycobacterium bovis* bacillus Calmette-Guérin (BCG) vaccine strain^13^. Removal of the RD1 locus in *M. tuberculosis* results in attenuated virulence, diminished intracellular growth^14,15^ and impaired granuloma formation in vivo^14–16^. Similar studies have highlighted a role for RD in the rupture of phagosomal membranes that facilitate cytosolic translocation in *M. tuberculosis*-infected cells^17,18^, as well as in the induction of type I interferons (IFNs) in bone-marrow derived macrophages *in vitro*^19,20^. While ESX-1 plays a key role in regulating the function of myeloid cells in vitro, its impact on monocyte transcription during in vivo infection remains unclear.

*M. marinum* is a close genetic relative to *M. tuberculosis* that is used as a model system to study mycobacterial pathogenesis^21,22^. Due to its low optimal growth temperature^23^, *M. marinum* primarily infect cooler tissues such as the skin and extremities in both humans and mice^22,24^. Importantly, *M. marinum* causes latency and granulomatous infections that histologically resemble tuberculosis disease and shares with *M. tuberculosis* the highly conserved ESX-1 secretion system^21,22,25,26^. We recently demonstrated, in a mouse model of *M. marinum* infection, that infiltrating monocytes and neutrophils represent the major bacteria-harboring cell populations in the infected tissue^10^, as well as an antagonistic interplay between these populations in disease pathology. We found that ESX-1 was essential in driving neutrophil recruitment into the infected tissue and their transition into a more proinflammatory phenotype. In contrast, infiltrating monocytes exhibited a host-protective role, dependent on the inducible nitric oxide synthase activity, by restraining ESX-1-dependent neutrophilic accumulation and immunopathology^10^.

We and others have characterised several mycobacterial transposon mutants deficient for different genes within the ESX-1 locus^27–30^. Such studies have suggested that the inactivation of ESX secretion-associated protein (Esp) K affects the secretion of Early Secreted Antigen Target 6 kDa (ESAT-6)^27–29,31^, a well-known immunogenic factor secreted by ESX-1. Analysis of infected bone marrow-derived macrophages revealed that *M. marinum* with a transposon insertion in *espK* (*espK*::Tn) retains its ability to permeabilize phagosomal membranes, translocate to the cytosol and promote IL1β production but, in contrast to WT *M. marinum*, fails to trigger type I IFN production^29^. Since pathogenic mycobacteria are thought to exploit type I IFN responses to promote disease^32–34^, it was of interest to explore the *espK*::Tn mutant in vivo.

In the current study we utilized the murine model of *M. marinum* infection in combination with single cell RNA sequencing (scRNA-seq) to assess the impact of ESX-1 on disease pathology and the transcription profile of infiltrating infected and bystander monocytes.

## Results

### Disease development and neutrophil and monocyte accumulation at sites of *M. marinum* infection are dependent on ESX-1 but independent of *espK* inactivation

**Fig. 1:**
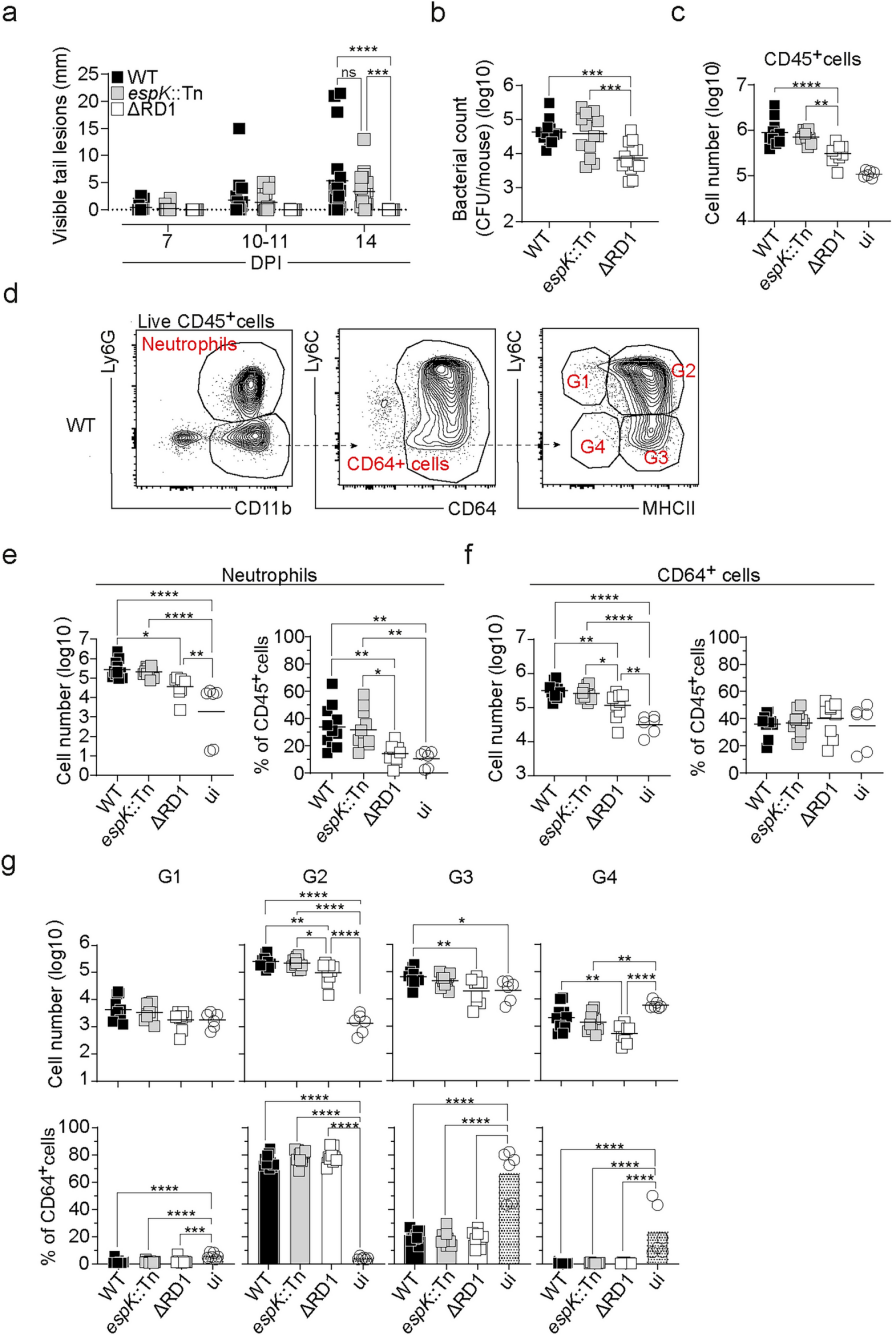
Disease development and neutrophil and monocyte accumulation at sites of *M. marinum* infection are dependent on ESX-1 but independent of *espK* inactivation. (**a**–**g**) C57BL/6 mice were infected with WT*, espK*::Tn or ΔRD1 *M. marinum*, or left uninfected (ui), as indicated. (**a**) Total length of visible tail lesions at indicated time points. Results are from 18 to 20 mice/group from three independent experiments. (**b**) Bacterial burden in tail draining (sciatic and inguinal) lymph nodes. Results are from 14 mice/group from 3 independent experiments. (**c**–**g**) Tails from infected mice were digested at 14 dpi and cell suspensions were stained and analyzed by flow cytometry. (**c**) Total number of Live CD45^+^ cells. (**d**) Representative flow cytometry gating and (**e** and** f**) total number and proportions of (**e**) CD11b^+^Ly6G^+^ neutrophils, (**f**) CD64^+^ monocyte and macrophages, and (**g**) monocyte/macrophage subsets as defined in G1 to G4 gates in (**d**) in tail cell suspensions of indicated mice. Results are from 6 uninfected or 9–11 infected mice/group pooled from 2 independent experiments. (**a**–**c**, **e–g**) Bar, mean. *p < 0.05, **p < 0.01, ***p < 0.001, ****p < 0.0001, as assessed by (**a**) two way or (**b–c, e–g**) one-way ANOVA.

To investigate the impact of the ESX-1 secretion system on *M. marinum* infection, C57BL/6 mice were infected with wildtype (WT) *M. marinum*, an *espK*::Tn mutant or an ΔRD1 mutant strain lacking the entire RD1 locus, via tail vein injection as previously described^10,35^. In this model, infection is selectively established in the mouse tail due to its reduced temperature^10,22^. WT infected mice had significantly increased tail lesions compared to ΔRD infected mice at 14 days post infection (dpi) however no differences in tail lesions were observed between WT and *espK*::Tn infected mice (Fig. 1a). As we wished to assess innate immune cell composition in the tails of infected mice (see below), we assessed bacterial load in tail-draining lymph nodes (sciatic and inguinal), that we have previously demonstrated mirrors that in the infected tail tissue^10^. While all strains could be detected in the tail draining lymph nodes at 14 dpi, the ΔRD1 strain was detected in significantly lower numbers compared with the WT and *espK*::Tn strains (Fig. 1b). Collectively these results confirm and extend our previous findings suggesting that *M. marinum* survival and pathology are ESX-1 dependent^10,22^, but unaffected by the transposon insertion in *espK*.

To assess whether infection with the ΔRD1 and *espK*::Tn mutants altered neutrophil and CD64^+^ myeloid cells accumulation, tails were digested and cell suspensions analyzed by flow cytometry, as previously described^10,35^. WT and *espK*::Tn infected mice had higher total numbers of CD45^+^ cells in infected tails compared to ΔRD1 infected mice (Fig. 1c). Similarly, the total number of neutrophils as well as the proportion of neutrophils amongst CD45^+^ cells was significantly lower in ΔRD1 compared with WT or *espK*::Tn infected mice (Fig. 1d and e). Total CD64^+^ myeloid cell numbers were also significantly lower in ΔRD1 compared with WT or *espK*::Tn infected mice although the proportions of CD64^+^ cells amongst CD45^+^ cells remained unchanged between infections (Fig. 1d and f). The CD64^+^ myeloid cell compartment can be divided into Ly6C^+^MHCII^−^ (gate 1 (G1)), Ly6C^+^MHCII^+^ (G2), Ly6C^−^MHCII^+^ (G3) and Ly6C^−^MHCII^−^ myeloid cells (G4) (Fig. 1d). As expected^3,10^, there was a marked increase in the number and proportion of Ly6C^+^MHCII^+^ monocytes in the tails of all infected mice (Fig. 1g), and Ly6C^+^MHCII^+^ monocyte numbers were slightly, yet significantly, reduced in ΔRD1 compared to WT or *espK*::Tn mutant infected mice (Fig. 1g). These findings suggest that ESX-1 drives neutrophil and monocyte accumulation to sites of *M. marinum* infection in a manner that is independent of *espK* inactivation.

### *espK*::Tn does not affect the ability of* M. marinum* to infect neutrophils and Ly6C^+^MHCII^+^ monocytes in vivo

**Fig. 2:**
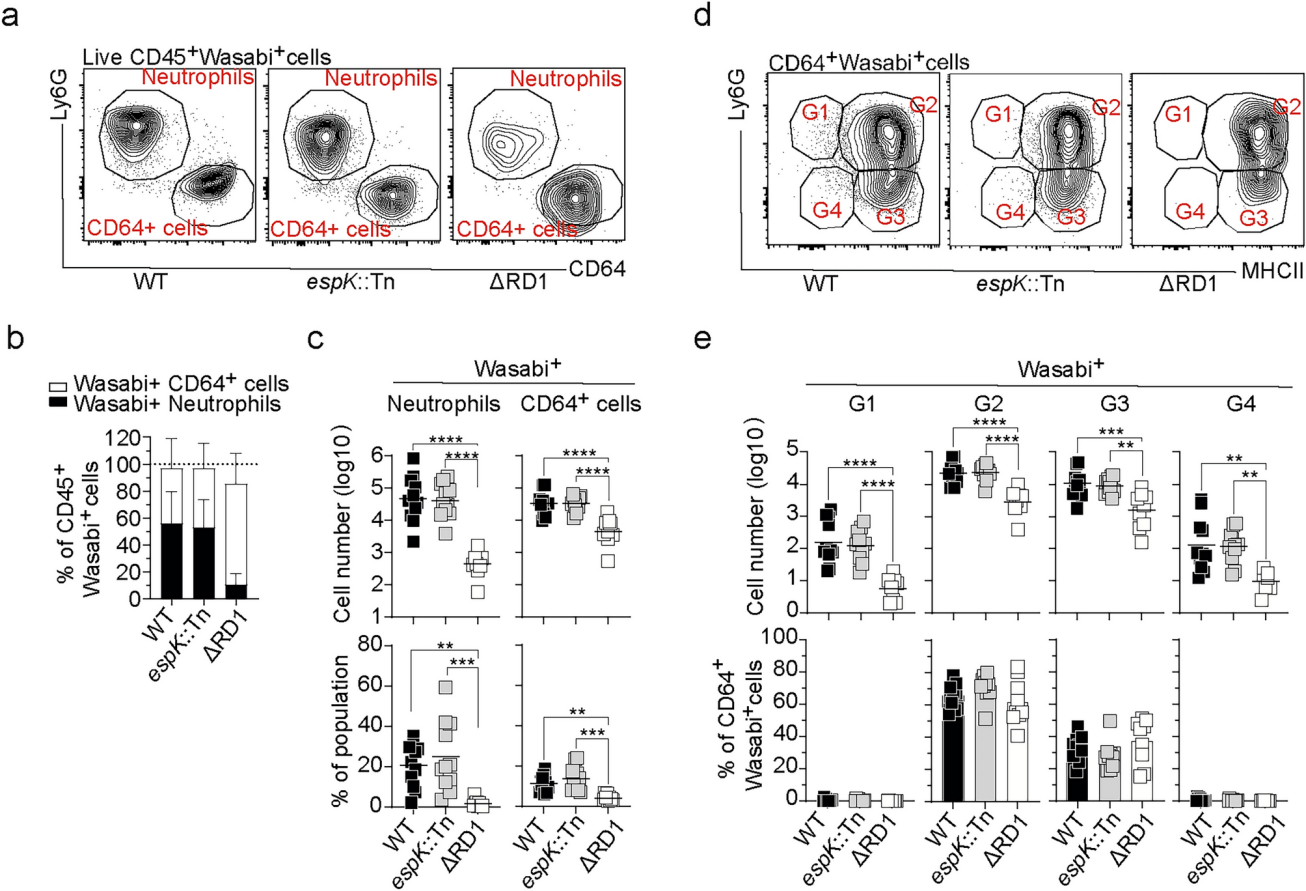
*espK*::Tn does not affect the ability of *M. marinum* to infect neutrophils and Ly6C^+^MHCII^+^ monocytes in vivo. C57BL/6 mice were infected with Wasabi-expressing WT, *espK*::Tn or ΔRD1 *M. marinum*. Tails from infected mice were digested at 14 dpi and cell suspensions were stained and analyzed by flow cytometry. (**a**) Representative flow cytometry plots showing Ly6G and CD64 expression on live CD45^+^ Wasabi^+^ cells, (**b**) proportion of Wasabi^+^ neutrophils and CD64^+^ cells among total Wasabi^+^ CD45^+^ cells and (**c**) number of Wasabi^+^ neutrophils and CD64^+^ cells (top panels) and proportion of Wasabi^+^ cells amongst total neutrophils or CD64^+^ cells (bottom panels). (**d**) Representative flow cytometry plots of Wasabi^+^ CD64^+^ within the G1 to G4 gates, (**e**) total number (top panels) and proportion (bottom panels) of Wasabi^+^ G1-G4 cells. (**a-e**) Results are from 9 to 11 mice/group and from two independent experiments. (**b**) Error bars, SD (**c**, **e**) Bar, mean. *p < 0.05, **p < 0.01, ***p < 0.001, ****p < 0.0001 as assessed by one-way ANOVA.

We have previously demonstrated that neutrophils and Ly6C^+^MHCII^+^ monocytes are the major bacteria-harboring cell types in the tail of both WT and ΔRD1 infected mice^10^. To confirm these findings and to determine the major infected cell populations in *espK*::Tn infected animals, we injected mice with WT, *espK*::Tn and the ΔRD1 strain expressing the strong green fluorescent protein “Wasabi”. Neutrophils and CD64^+^ myeloid cells represented the main bacteria-harboring cell types in WT and ΔRD1 infected mice and a similar pattern was observed in *espK*::Tn infected mice (Fig. 2a). Collectively, infected neutrophils and CD64^+^ myeloid cells made up approximately 100% of all Wasabi-positive CD45^+^ cells in all three infections (Fig. 2b). While ΔRD1 primarily infected CD64^+^ myeloid cells, WT and *espK*::Tn showed a higher ratio of infected neutrophils (Fig. 2b), which likely reflects the simultaneous ESX-1-mediated accumulation of neutrophils in the tissue (Fig. 1e). Overall, WT and *espK*::Tn infected mice had significantly higher numbers and proportions of infected neutrophils and CD64^+^ myeloid cells compared to ΔRD1 infected mice (Fig. 2c). Detailed analysis of the CD64^+^ myeloid cell population showed that Ly6C^+^MHCII^+^ monocytes were the main bacteria-harboring subset in all three infections (Fig. 2d–e). These results indicate that *M. marinum* primarily infects neutrophils and infiltrating Ly6C^+^MHCII^+^ monocytes in a manner that is independent of *espK* inactivation.

### *M. marinum* infection alters the transcriptional profile of Ly6C^+^MHCII^+^ monocytes

**Fig. 3:**
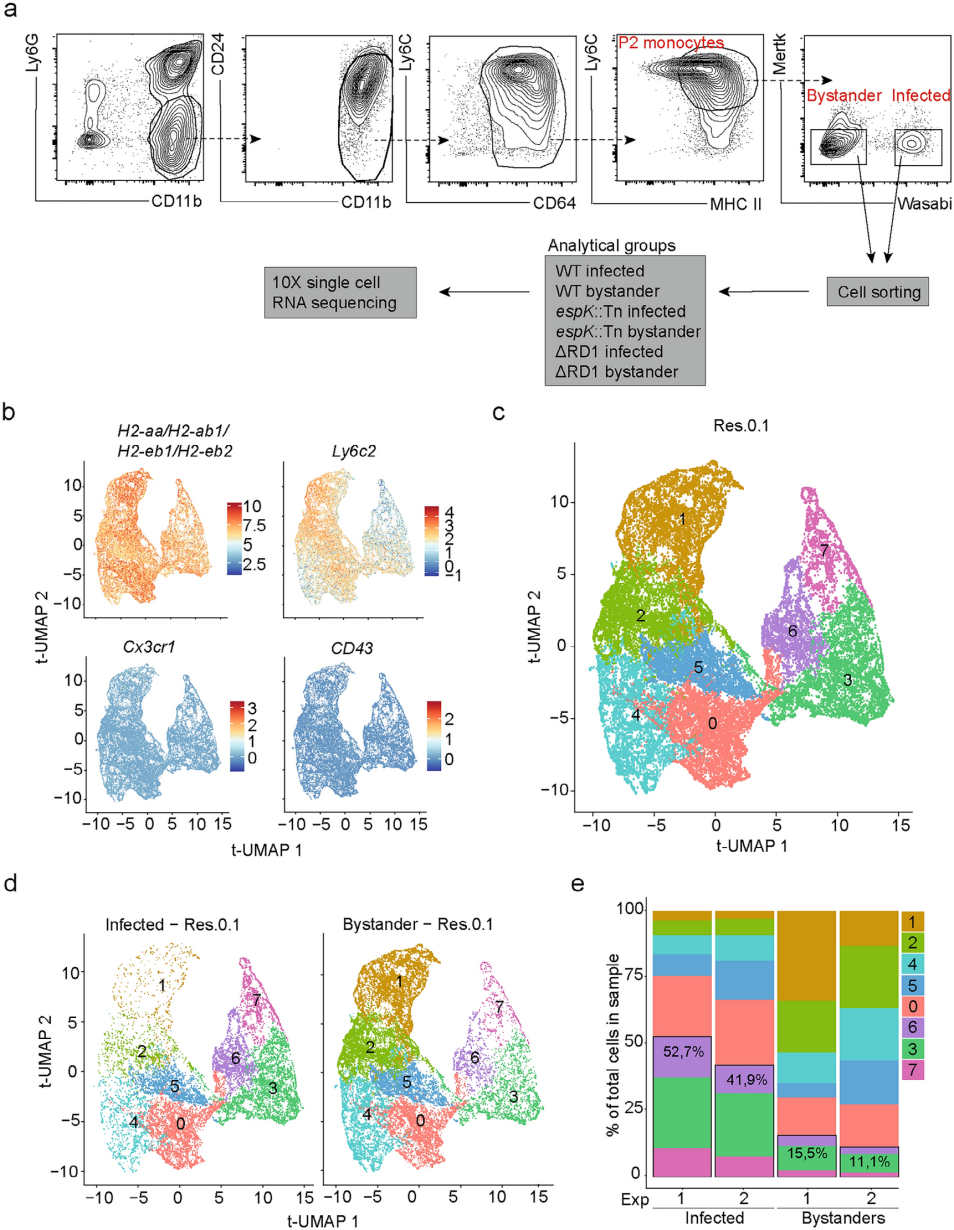
*M. marinum* infection alters the transcriptional profile of Ly6C^+^MHCII^+^ monocytes. (**a**–**e**) Mice were infected with Wasabi-expressing WT, *espK*::Tn or ΔRD1 *M. marinum* and infected (Wasabi^+^) and bystander (Wasabi^−^) Ly6C^+^MHCII^+^CD64^+^ myeloid cells were sorted form infected tails 14 dpi and subjected to scRNA-seq. (**a**) Representative flow cytometry gating for the sorting of infected and bystander myeloid cells. (**b**) Expression of indicated genes overlayed on Trajectory based nearest neighbor analysis (t-Space) UMAP (tUMAP) from the 11 scRNA-seq samples pooled from 2 different experiments (*espK*::Tn and ΔRD1 Wasabi^+^ and WT, *espK*::Tn and ΔRD1 Wasabi^−^ from experiment 1, and WT, *espK*::Tn and ΔRD1 Wasabi^+^ and WT, *espK*::Tn and ΔRD1 Wasabi^−^ from experiment 2). (**c**) tUMAP depicting eight clusters identified at 0.1 resolution. (**d**) tUMAP visualization of infected (Wasabi^+^, left panel) and bystander (Wasabi^−^, right panel) Ly6C^+^MHCII^+^CD64^+^ myeloid cells. (**e**) Cluster proportions are shown for infected samples (*espK*::Tn and ΔRD1 Wasabi^+^ for experiment 1, and WT, *espK*::Tn, and ΔRD1 Wasabi^+^ for experiment 2) in the left bars, and for bystander samples (WT, *espK*::Tn, and ΔRD1 Wasabi^−^ for experiment 1, and WT, *espK*::Tn, and ΔRD1 Wasabi^−^ for experiment 2) in the right bars of the graph.

Because Ly6C^+^MHCII^+^ monocytes are the major infected CD64^+^ myeloid population in vivo (Fig. 2e), we next assessed the impact of ESX-1 on the transcriptional profile of these cells. To this end, mice were infected with Wasabi-expressing WT, *espK*::Tn and ΔRD1 *M. marinum,* and Wasabi^+^ and Wasabi^−^ CD64^+^Ly6C^+^MHCII^+^ monocytes were sorted from the infected tails by flow cytometry at 14 dpi and subject to scRNA-seq (Fig. 3a). CD64^+^Ly6C^+^MHCII^+^ monocytes are barely present in the tails of uninfected mice, restricting our analysis to Wasabi^+^ and Wasabi^-^ monocytes sorted from infected tails. Of note, while the bystander (Wasabi^−^) cells are not infected at the time of analysis, it cannot be excluded that they may have cleared an infection or internalized mycobacterial debris prior to analysis, all of which could have influenced their transcriptional profiles. Samples were obtained from two independent experiments. One sample (WT infected [Wasabi^+^] from experiment 1) was excluded from further analysis due to a low cell count and low gene yield/cell. The remaining 11 samples were bioinformatically pooled after removal of contaminants and visualized on a trajectory based nearest neighbor analysis UMAP (tUMAP)^36^. Cells from both experiments were similarly represented in the tUMAP (Supplementary Fig. S1) and as expected expressed MHC class II associated genes and *Ly6c*, but lacked expression of the non-classical monocyte associated genes *Cx3cr1* and *CD43* (Fig. 3b)^37^.

Eight interlinked clusters could be identified within the trajectory based nearest neighbor analysis at resolution 0.1 (Fig. 3c). The size of each of these clusters differed markedly between infected and bystander Ly6C^+^MHCII^+^ monocytes (Fig. 3d), with clusters 3, 6 and 7 dominating amongst infected cells and clusters 1, 2 and 4 dominating amongst uninfected cells across both experiments (Fig. 3d–e). Thus *M. marinum* infection alters the transcriptional profile of Ly6C^+^MHCII^+^ monocytes in vivo (Fig. 3d–e).

### *M. marinum* infection induces a metabolic shift and alterations in chemokine/cytokine transcription in Ly6C^+^MHCII^+^ monocytes in vivo

**Fig. 4:**
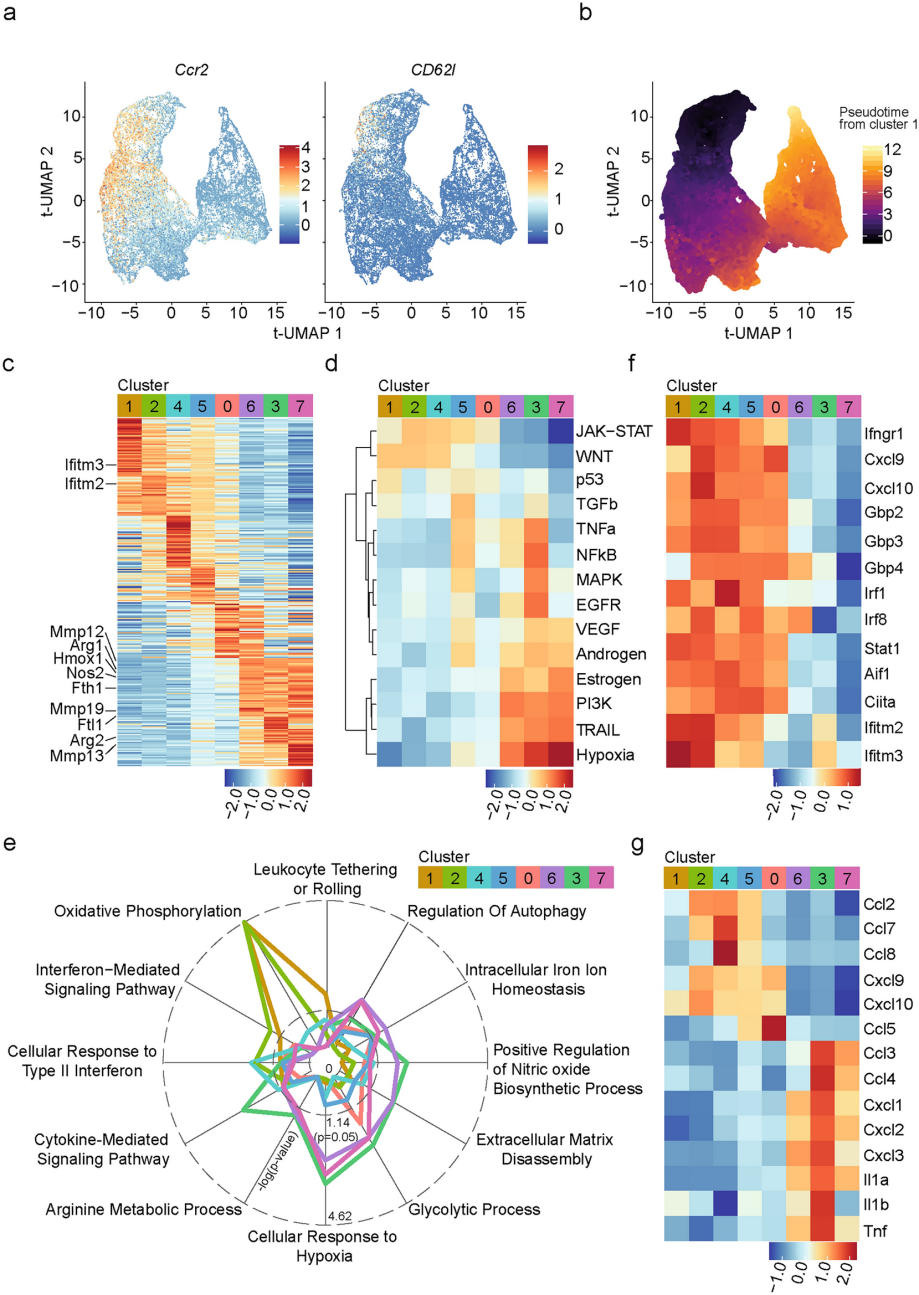
*M. marinum* infection induces a metabolic shift and alterations in chemokine/cytokine transcription in Ly6C^+^MHCII^+^ monocytes in vivo. (**a–f**) Transcriptional characterization of Ly6C^+^MHCII^+^CD64^+^ clusters. (**a**) Expression of indicated genes overlayed on tUMAP. (**b**) Pseudotime overlay on tUMAP of scRNA-seq data obtained from infected and bystander Ly6C^+^MHCII^+^ CD64 + myeloid cells using cluster 1 as the pseudotime starting point. (**c**) Scaled heatmap showing the average expression of the top 50 Differentially expressed genes (DEGs) per cluster. (**d**) Signaling pathway activity within individual clusters as assessed using PROGENy^32–34^. (**e**) Radar chart showing selected enriched pathways within indicated clusters using Gene Ontology Biological Process 2023 (Enrichr) tool. (**f** and** g**) Scaled heatmap showing the average expression of selected genes within (**f**) the *Cellular Response to Type II Interferon* or *Interferon-Mediated Signaling Pathway*, and (**g**) the *Cytokine-Mediated Signaling Pathway*, in indicated clusters.

A proportion of cells within cluster 1 expressed both *Ccr2* and *CD62l* (Fig. 4a), markers involved in monocyte trafficking and infiltration into infected tissues^38^, indicating that this cluster represented the earliest monocyte derived population in our data set. Pseudotime analysis using cluster 1 as a starting point indicated a developmental trajectory from cluster 1, through cluster 0 and towards cluster 3, 6 and 7 (Fig. 4b). Differentially expressed gene (DEG) analysis identified cluster-specific genes whose expression patterns appeared to align with the predicted pseudotime analysis creating a gradient expression pattern of DEGs from cluster 1 to cluster 7 (Fig. 4c). Based on these transcriptional transitions, we defined clusters 1, 2 and 4 as “early clusters”, cluster 5 and 0 as “intermediate clusters” and cluster 3, 6 and 7 as “late clusters”, a terminology referring to the transcriptional trajectory of CD64^+^Ly6C^+^MHCII^+^ cells in the infected tissue. Analysis of signaling pathway activity using the Pathway RespOnsive GENes (PROGENy) package^39–41^, suggested high activation of JAK-STAT pathways, that are associated with cytokine and interferon stimulation^42^, and WNT signaling that is associated with phagocytic activity in macrophages^43^, in early clusters (Fig. 4d). In contrast, late clusters 3 and 6, along with intermediate cluster 5, showed higher activation of TNFa, NFkB, MAPK and EGFR signaling pathways (Fig. 4d), which are involved in regulating immune responses, cell differentiation and survival. Late clusters 3, 6 and 7 also showed upregulation of hypoxia, TNF-related apoptosis inducing ligand (TRAIL) and PI3K signaling pathways (Fig. 4d). TRAIL is a potent regulator of extrinsic apoptosis in immune cells, but it has also been shown to induce non-apoptotic signaling pathways, including NFkB, P13K and MAPK^44^.

To identify potential pathways that are up- or downregulated in Ly6C^+^MHCII^+^ monocytes as they differentiate within the infected tissues, we performed Gene Ontology (GO) enrichment analysis of the DEGs within each cluster (Fig. 4e). Cluster 1 was enriched for genes associated with the *Leukocyte Tethering or Rolling* pathway consistent with the idea that these cells represent the earliest monocyte derived population in our data set (Fig. 4e). Early clusters 1 and 2 were enriched for genes associated with *Oxidative Phosphorylation* and *Interferon-Mediated Signaling* while early clusters 2 and 4 were enriched for genes associated with the *Cellular Response to Type II Interferon* (Fig. 4e). Indeed, early and intermediate clusters expressed many IFNγ-induced genes at higher levels than late clusters (Fig. 4f), suggesting that infiltrating monocytes may rapidly respond to IFNγ upon arrival within the tissue.

In contrast, late clusters were enriched in genes associated with *Glycolytic Process, Positive Regulation of Nitric Oxide Biosynthetic Process, Arginine Metabolic Process, Cellular Response to Hypoxia, Extracellular Matrix Disassembly*, *Intracellular Iron Ion Homeostasis,* and *Regulation of Autophagy* (Fig. 4e). Interestingly both early cluster 4 and late cluster 3 showed enrichment for the *Cytokine-Mediated Signaling* pathway (Fig. 4e), and further analysis demonstrated a clear dichotomy in the expression of chemokines and cytokines between early/intermediate and late clusters (Fig. 4g).

Collectively, these data suggest that *M. marinum* infection drives major transcriptional changes in Ly6C^+^MHCII^+^ monocytes, including a metabolic shift towards glycolytic metabolism, a switch in chemokine/cytokine production, and reduced responsiveness to IFNγ signaling.

### *M. marinum* infection of Ly6C^+^MHCII^+^ monocytes alters the transcription profile of individual early and intermediate clusters

**Fig. 5:**
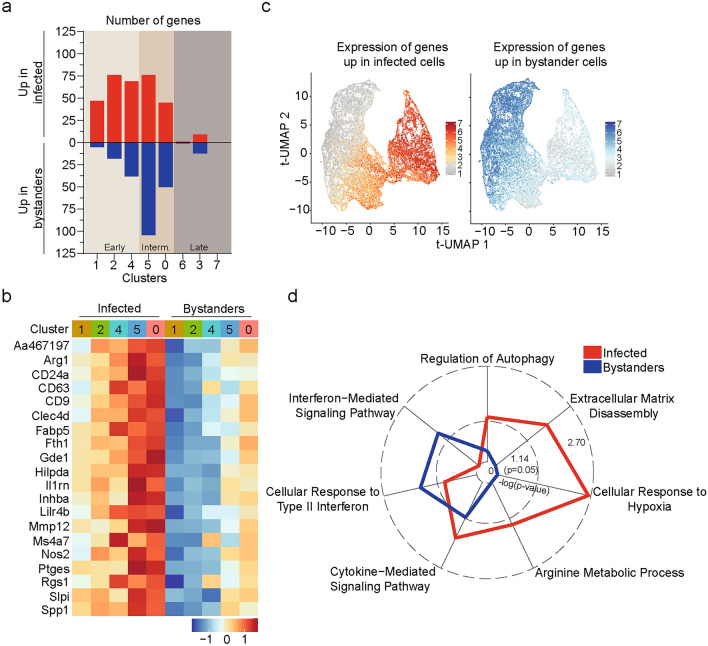
*M. marinum* infection of Ly6C^+^MHCII^+^ monocytes alters the transcription profile of individual early and intermediate clusters. (**a**–**d**) Transcriptional analysis of infected versus bystander Ly6C^+^MHCII^+^CD64^+^ myeloid cell clusters. (**a**) Graph depicting number of significantly up and down regulated genes between individual infected and bystander clusters. Differential expression was defined by an average log fold change > 0.5 and a p-value < 0.05, consistent across the two experiments. (**b**) Scaled heatmap showing the average expression of genes that were commonly enriched in infected cells across all early and intermediate clusters. (**c**) Expression level of the 114 genes significantly upregulated in infected clusters (left panel) and the 124 genes significantly upregulated in bystander cells (right panel) calculated using AddModuleScore^68^ and overlayed on the scRNA-seq based tUMAP. (**d**) Radar chart showing selected signaling pathways enriched infected and bystander cells using Gene Ontology Biological Process 2023 (Enrichr) tool.

We next assessed the impact of infection on the transcriptional profile of individual clusters using an average log fold change > 0.5 and p-value < 0.05 in both independent experiments as a selection criterion. Infected and bystander cells differed in their expression of 238 genes with 114 genes significantly enriched in infected cells and 124 genes significantly enriched in bystander cells (Supplementary Table S1). Most differences in gene expression between infected and bystander cells were observed in the early and intermediate clusters (Fig. 5a). Twenty of the genes that were enriched in infected cells were common across all early and intermediate clusters (Fig. 5b). Notably, genes enriched in infected early and intermediate clusters were primarily genes whose expression was associated with later clusters while genes higher in bystander early and intermediate clusters were primarily associated with early clusters (Fig. 5c). Consistent with this finding, GO enrichment analysis demonstrated that genes enriched in infected cells within early and intermediate clusters were associated with *Cellular Response to Hypoxia, Extracellular Matrix Disassembly*, *Arginine Metabolic Process* and *Cytokine-Mediated Signaling* (Fig. 5d), while bystander cells within early and intermediate clusters were associated with *Interferon-Mediated Signaling* and *Cellular Response to Type II Interferon* pathways (Fig. 5d). Thus, in addition to changing the proportion of individual clusters, infection impacted on the transcriptional profile of early and intermediate clusters appearing to initiate their transition towards the late clusters.

### ESX-1 drives differentiation of bystander Ly6C^+^MHCII^+^ monocytes into late clusters

**Fig. 6:**
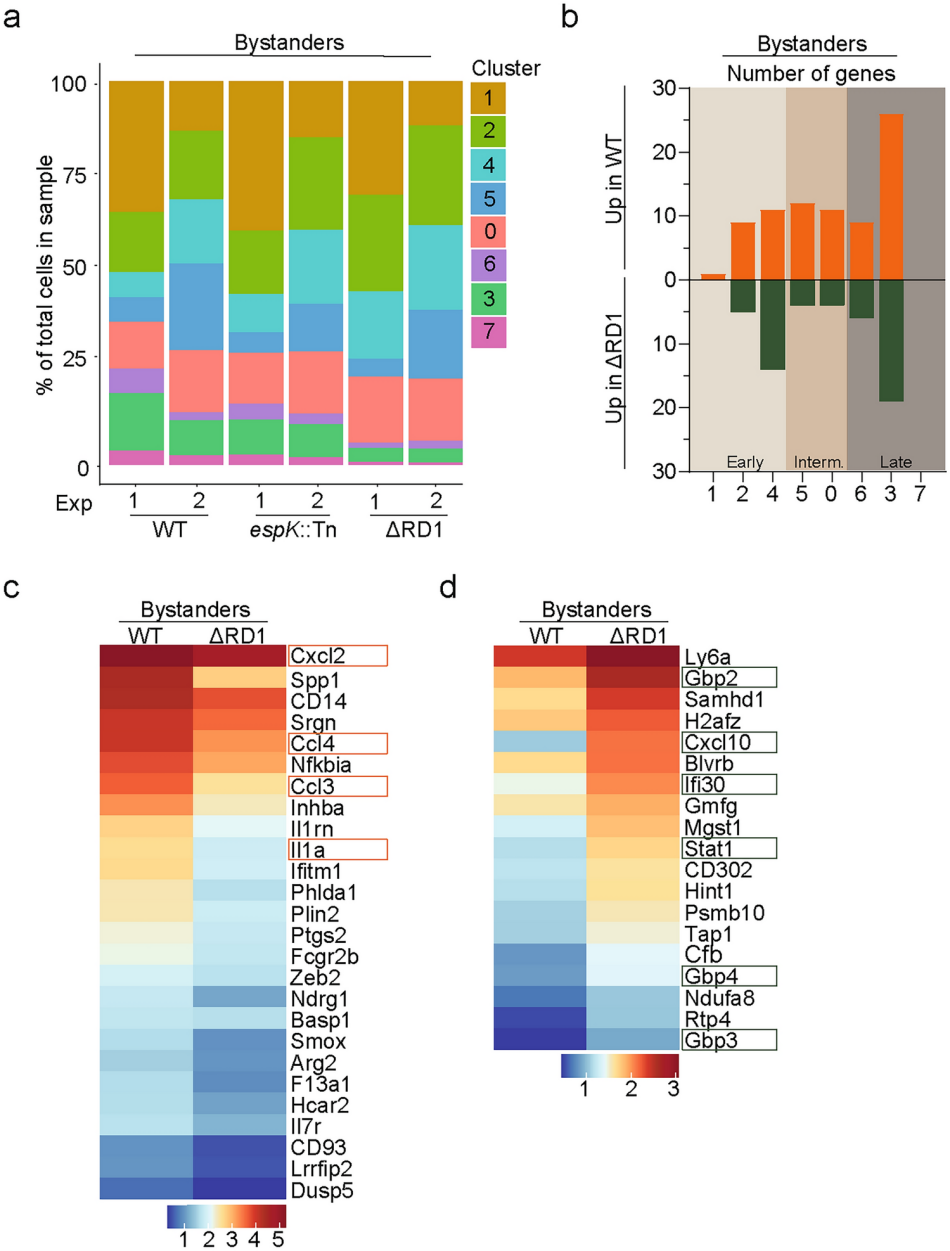
ESX-1 drives differentiation of bystander Ly6C^+^MHCII^+^ monocytes into late clusters. (**a**–**d**) Transcriptional analysis of Ly6C^+^MHCII^+^CD64^+^ myeloid cells, comparing bystander cells from WT versus ΔRD1 *M. marinum* infection. (**a**) Proportions of individual clusters among bystander cells from WT, *espK*::Tn or ΔRD1 *M. marinum* infected mice from indicated experiments. (**b**) Number of up and down regulated genes in individual bystander clusters from WT and ΔRD1 infected mice. (**c** and** d**) Non-scaled heatmap showing the average expression of DEGs within cluster 3, for (**c**) DEGs enriched in WT bystander cells, and (**d**) DEGs enriched in ΔRD1 bystander cells, in indicated samples. (**b**–**d**) Differential expressions were defined by an average log fold change > 0.5 and a p-value < 0.05, consistent across the two experiments.

To assess the impact of ESX-1 on the transcriptional profile of Ly6C^+^MHCII^+^ monocytes we first compared the sizes of clusters between WT, *espK*::Tn and ΔRD1 infected mice. As only one replicate of the WT infected sample was available, we focused our analysis on the bystander populations. The proportion of cluster 1 cells in the bystander population differed between experiment 1 and 2 (Fig. 6a), however this was independent of bacterial strain and likely reflects minor differences in gating stringency during cell sorting between the two experiments. While the proportions of cells within late clusters were lower in bystander compared to infected populations (Fig. 3e), ΔRD1 infected mice had a smaller proportion of bystander Ly6C^+^MHCII^+^ cells within late clusters compared to WT and *espK*::Tn infected mice across both experiments (Fig. 6a). These results indicate that ESX-1 promotes the transition of bystander Ly6C^+^MHCII^+^ monocytes towards later clusters, independent of the transposon insertion in *espK*.

We next explored whether individual clusters might exhibit transcriptional differences depending on whether they came from WT, *espK*::Tn and ΔRD1 infected mice. The transcription profile of bystander clusters did not differ between WT and *espK*::Tn infections. In contrast, bystander cells from WT and ΔRD1 infected mice differed in the expression of 78 genes across all clusters, with 42 genes significantly higher in bystander cells from WT infected mice and 36 genes significantly higher in bystander cells from ΔRD1 infected mice (Supplementary Table S2). Differences in gene expression were observed throughout the trajectory, with the highest differences of 45 genes occurring in late cluster 3 (Fig. 6b). In cluster 3, bystander Ly6C^+^MHCII^+^ monocytes from WT infected mice were enriched for several cytokines, including *Il1a*, *Ccl3*, *Ccl4* and *Cxcl2* (Fig. 6c). In contrast, ΔRD1 bystander monocytes were enriched for genes associated with IFNγ-mediated signaling, including *Stat1*, *Cxcl10*, Guanylate binding protein 2 (*Gbp2*), *Gbp3*, *Gbp4* and *Ifi30.* (Fig. 6d). Collectively these results suggest a role for ESX-1 in evading IFNγ-mediated immunity by regulating the phenotype of bystander monocytes.

## Discussion

In the current study, we explored the role of the ESX-1 secretion system in regulating *M. marinum* infection and myeloid cell function in vivo. We find that infection of recruited monocytes markedly alters their transcriptional profile in the infected tissue. We further demonstrate that the transcriptional profile of bystander monocytes is influenced by ESX-1. Taken together, our studies provide novel insights regarding the regulation of myeloid cell function in the setting of mycobacterial infection.

As expected, monocytes represented a major infected cell population in the tails of mice during infection with all three *M. marinum* strains. While total as well as infected monocyte numbers were reduced in ΔRD1 mutant compared with WT infected mice, the proportions of each of the four CD64^+^ myeloid cell subsets appeared similar across infections. Thus, at the infectious dose used, ESX-1 appears to be required for optimal *M. marinum-*induced monocyte recruitment and/or maintenance, but not for regulating the transition of infiltrating monocytes into Ly6C^+^MHCII^+^ and Ly6C^−^ MHCII^+^ cells. These findings contrast with our previous study where mice infected with a tenfold lower infectious dose of WT and ΔRD1 showed no significant differences in numbers of CD64⁺ cells or its subpopulations (G1-G4) at 14 dpi^10^. Consistent with this apparent dose-dependent difference, it has been shown that the infectious dose of *M. tuberculosis* affects monocyte recruitment and function^11^, and a recent study demonstrates that it influences macrophage function with subsequent effects on T cell responses^45^.

To assess more broadly the impact of *M. marinum* infection on monocyte transcription, we performed scRNA-seq on Ly6C^+^MHCII^+^ monocytes, the numerically largest populations of infected CD64^+^ myeloid cells in all three infections. Such analysis demonstrated that infection drove a metabolic shift in these cells from oxidative metabolism to glycolysis, an upregulation in nitric oxide synthesis, iron ion metabolism, and an enhanced responsiveness to hypoxia mediated signaling. These findings confirm and extend previous studies in *M. tuberculosis* infected mouse lung indicating that infection induces enhanced glycolysis, HIF1α-mediated signaling, production of reactive oxygen species, NOS2 expression and iron metabolism in interstitial lung macrophages^46,47^. Such infection driven changes have been associated with bacterial growth restriction^6,46,47^. Similarly, *M. tuberculosis* infection of human monocyte-derived macrophages promotes glycolysis and IL-1β responses, both of which have been associated with bacterial growth control^48^. Consistent with this, we also noted upregulation of arginine metabolic processes including induction of Arginase 1 (*Arg1*) and *Arg2* that metabolizes arginine independently of oxygen and has been shown to control *M. tuberculosis* growth in granulomas when NOS2 is rendered ineffective by hypoxia^49^. Collectively these results suggest that mycobacterial infection of Ly6C^+^MHCII^+^ monocytes induce transcriptional changes that create a less permissive environment for bacterial growth.

We also noted marked differences in the expression of several chemokines, cytokines and Matrix metalloproteinases (MMPs) between infected and bystander Ly6C^+^MHCII^+^ monocytes. For example, bystander cells were enriched for IFNγ-mediated signaling, indicating that mycobacterial infection suppresses responsiveness to IFNγ in a cell intrinsic manner, an observation consistent with previous findings in bone-marrow derived macrophages infected with pathogenic mycobacteria^50–53^*.* Infection drove marked alterations in chemokine transcription including a downregulation of the CCR2 ligands *Ccl2* and *Ccl7* and the IFNγ inducible CXCR3 ligands *Cxcl9* and *Cxcl10*. Since CCL2 and CCL7 regulate monocyte recruitment and T-cell activation and CXCL9 and CXCL10 the recruitment of CXCR3^+^ T-cells during mycobacterial infection^7–11,54–56^, our results suggest that bystander monocyte populations may play a more important role in these processes compared to their infected counterparts. In contrast, *M. marinum* infection of Ly6C^+^MHCII^+^ monocytes lead to increased transcription of *Il1a*, *Il1b* and *Tnf* as well as several MMPs. Since TNF together with MMPs controls several aspects of *M. tuberculosis* infection, including granuloma formation^57–60^, our results suggest a more dominant role for infected rather than bystander Ly6C^+^MHCII^+^ monocytes in this process. Collectively our findings suggest that infected and bystander Ly6C^+^MHCII^+^ monocytes may play distinct roles in regulating immune cell recruitment and disease pathology during *M. marinum* infection.

WT and *espK*::Tn *M. marinum* were found in similar numbers in the tail draining lymph nodes of infected mice, consistent with findings that EspK deficiency does not impact *M. tuberculosis* replication in the lung and spleen^31^. We find that disease pathology, cellular recruitment and the cluster proportions and transcriptional profile of bystander Ly6C^+^MHCII^+^ cells was indistinguishable between WT and *espK*::Tn infected mice. These results contrast with our prior observation that that the *espK*::Tn mutant, unlike WT, fails to induce type I IFNs in bone marrow-derived macrophages in vitro^29^, and suggests that EspK deficiency plays a minor role in *M. marinum* virulence and pathology in vivo. While further studies are required to dissect underlying mechanisms, one possibility may be that the mechanisms driving type I IFN induction differ between infected macrophages in vitro and monocytes or other cell types in vivo. Consistent with this hypothesis, it is known that *Streptococcus pyogenes* induces type I IFN production via different mechanisms in macrophages (STING-dependent) and dendritic cells (IRF5-dependent)^61^.

Multiple studies have demonstrated that ESX-1 has significant effects on the transcriptional profile of infected myeloid cells *in vitro*^19,20,29,51^. A recent analysis of human monocyte-like THP-1 cells, however, indicate similar transcriptional responses to infection with *M. tuberculosis* and BCG, which lacks ESX-1^13^, including the upregulation of genes involved in the immune response and cytokine signaling pathways^62^. Nevertheless, *M. tuberculosis* induced greater transcriptional changes in THP-1 cells than BCG, in particular early during the infection, while BCG triggered apoptosis^62^. While the role of ESX-1 in regulating myeloid cell transcription in vivo remains less clear, infection of mice with a BCG vaccine strain was recently found to induce similar epigenetic and transcriptional changes in alveolar and interstitial lung macrophages as *M. tuberculosis*^47^. Thus, it appears that much of the transcriptional response in infected monocytes in vivo is driven by conserved host–pathogen interaction, with strain-specific modulations rather contributing to fine-tuning these responses. Consistent with this idea,  while the transcriptional profile of bystander Ly6C^+^MHCII^+^ cells differed between WT and ΔRD1 *M. marinum* infected mice differences were limited.

Bystander Ly6C^+^MHCII^+^ cells from WT infected mice expressed higher levels of *Il1a*, *Ccl3/4* and *Cxcl2* and decreased levels of genes associated with IFNγ-mediated signaling, including the IFNγ-inducible GBPs, compared with bystander cells from ΔRD1 mutant. Since bystander cells represent the dominant CD64^+^ myeloid population in infected tissues, and the CXCL2 receptor CXCR2 is involved in neutrophil recruitment in *M. tuberculosis* infection^63,64^, we hypothesize that reduced expression of *Cxcl2* by bystander monocytes in ΔRD1 infected mice might, at least in part, underlie the reduced neutrophil numbers observed in these mice. The mechanism(s) underlying the different transcriptional profiles of bystander cells from WT and ΔRD1 infected mice remain unclear. While this might be partly due to a higher number of bystander cells in WT infected mice having previously encountered mycobacteria, either through cleared infections or by uptake of bacterial debris, we speculate that it likely reflects the different inflammatory environments established during these two distinct infections^10^. In this regard, we recently observed similar findings in neutrophils from *M. marinum* and ΔRD1 infected mice^10^. Thus, while ESX-1 has limited impact on the transcriptional profile of infected neutrophils, bystander neutrophils adopted a proinflammatory phenotype in an ESX-1-dependent manner^10^.

In summary, the current study provides novel insights into monocyte heterogeneity and the role of ESX-1 in regulating Ly6C^+^MHCII^+^ monocyte transcription during mycobacterial infection in vivo. Our results suggest that *M. marinum* alters the transcriptional profile of infected monocytes in the tissue and that the transcription response of bystander myeloid cells is influenced by ESX-1-dependent mechanisms, potentially contributing to disease pathology.

## Materials and methods

### Animals

C57Bl/6JRj mice were purchased from Janvier Labs and maintained at the Center for Comparative Medicine, Biomedical Centre (BMC), Lund University.

### Infections

Wild type *M. marinum* M strain, the isogenic mutant lacking the RD1 locus (ΔRD1)^25^ and the *espK* transposon insertion mutant (*espK*::Tn)^27^ were inoculated from frozen stocks in Middlebrook 7H9 medium (BD Biosciences) supplemented with glycerol (0.5%, Sigma-Aldrich), Tween 80 (0.05%, Sigma-Aldrich), Albumin-Dextrose-Catalase (ADC) Supplement (10%, BD Biosciences) and Hygromycin (50 µg/mL, Invitrogen). All strains carried the pTEC15 plasmid (Addgene) for green fluorescent ‘wasabi’ expression, with selection through Hygromycin B. Inoculated bacteria were grown under slow shaking conditions at 30 °C until late log phase and prepared for infection as previously described^35^. In short, bacteria were collected through centrifugation, washed twice in sterile PBS and passaged three times through a 26G needle, followed by two centrifugations at 500 g for 1 min to collect single-cell bacterial suspensions. Female mice (8–12 week old) were injected via the tail vein with bacteria, as previously described, but using a tenfold higher dose (7 × 10^7^ colony forming units (CFU) in 200 µL PBS/mouse), which in preliminary experiments was shown to enhance myeloid cell numbers in the tail of infected mice. Disease development was quantified by summing the length of individual tail lesions per mouse. The animals were euthanized at the indicated timepoint with increasing concentrations of carbon dioxide, followed by cervical dislocation to ensure death.

### Determination of bacterial loads in tissues

Tail draining lymph nodes (sciatic and inguinal lymph nodes) were collected and prepared for analysis as previously described^35^. Briefly, lymph nodes were homogenized in PBS containing Triton X (0.1%) using a stainless bead with the TissueLyser II (Qiagen). Homogenized tissues were serially diluted and plated on Middlebrook 7H10 agar (BD Biosciences) supplemented with glycerol (0.5%, Sigma-Aldrich), and Oleic acid Albumin-Dextrose-Catalase (OADC) (10%, Conda Lab). CFUs were determined after incubating the plates for 5 to 7 days at 30 °C.

### Flow cytometry analysis

Tails were collected and prepared for flow cytometry as previously described^35^. In brief, tails were severed from mice at the tail base and the skin was separated from the bone after a longitudinal excision. Tail skins were cut into 2 mm pieces, transferred into Dulbecco´s Modified Eagle Medium (DMEM) (Gibco™/LifeTechnologies) containing FCS (5%), Liberase TM (30 µg/mL, Roche) and DNAse I (52 µg/mL, Sigma), and incubated at 37 °C with magnetic stirring for 60 min. Samples were filtered through a nylon cell strainer (70 µm), washed in FACS buffer (PBS containing FCS (3%) and EDTA (2 mM)) and filtered through a nylon cell strainer (40 µm). Cell suspensions were incubated with rat anti-mouse CD16/CD32 antibody (2.4G2) to block Fc-receptors. Cell surface staining was performed in PBS for 30 min on ice with the fixable viable dye Near-IR Dead Cell Stain Kit (Invitrogen) and the following fluorochrome-conjugated anti-mouse antibodies: CD45.2 (104 or 30F-11), CD11b (M1/70), CD64 (X54-5/7.1), Ly6C (HK1.4), Ly6G (1A8), MHCII (M5/114.15.2), CD24 (M1/69), Mertk (2B10C42), CD19 (6D5), TCRβ (H57-597). Cells were then fixed with PFA (2%) for 20 min at room temperature. AccCount Fluorescent 20 Particles (Spherotech) counting beads were added to each sample to determine the number of cells per sample during flow cytometry analysis. Flow cytometry analyses were performed on an LSR II flow cytometer (BD Biosciences) and data was analyzed using FlowJo software version 10.

### Single-cell RNA-sequencing

Two independent experiments were conducted, with each experiment involving collection, pooling, and processing of 14 to 15 tails per group (bacterial strain), following the specified protocol for flow cytometry analysis. Using a FACS ARIA Fusion (BD Biosciences), Live CD45^+^ CD11b^+^CD64^+^Ly6C^+^MHCII^+^Mertk^-^Wasabi^+^ or Wasabi^−^ cells were sorted in PBS containing BSA (1%). Mock-infected controls could not be utilized in this model as tail tissue from uninfected mice contain insufficient numbers of Ly6C^+^MHCII^+^ “G2” cells. Single-cell RNA-sequencing libraries were prepared according to the manufacturer’s instructions. This process utilized the Chromium Single-cell 3′ Library & Gel Bead Kit v3 (10 × Genomics, PN-1000092) and Chromium Chip B Single-cell Kit (PN-1000074) in conjugation with the Chromium Controller & Next GEM Accessory Kit (10 × Genomics, PN-120223). In brief, single cells, reverse transcription reagents, barcoded Gel Beads, and oil were combined on a microfluidic chip to form Gel Beads in Emulsion (GEMs). Cells were lysed within GEMs, and released poly-A transcripts were barcoded with Illumina R1, a 10X barcode and a Unique Molecular Identifier (UMI) during the reverse transcription. The resulting barcoded cDNA were purified, amplified by PCR, and indexed sequencing libraries were constructed. Library quality control and quantification were performed using a KAPA Library Quantification Kit (Kapa Biosystems, KK4873) and the 2100 Bioanalyzer with a High Sensitivity DNA kit (Agilent, 5067-4626).

Illumina deep sequencing was performed on pooled indexed libraries by NextSeq 500/550 High Output v2.5 kit (150 cycles) at the Center of Excellence for Fluorescent Bioanalytics (KFB, University of Regenburg, Germany) or by Novaseq 6000 S1 or S2 (200 cycles) at the SNP&SEQ Technology Platform (Uppsala, Sweden) for each experiment, respectively.

### Bioinformatic analyses

The sequencing data was pre-processed and aligned using CellRanger, with versions 2.1.1 and 3.1.0 employed for each independent experiment, respectively^65^. Alignments were conducted with the reference genomes mm10 and mm10-2.1.0 for the respective experiments. Subsequently, the data underwent quality checking using R (version 4.0.2), involving the removal of cells with high mitochondrial gene content (> 10%) and/or very low number of reads and genes (< 430 genes per cell). Additionally, cells with very high gene counts (> 5500 genes per cell) were excluded as suspected doublets. Sample “WT infected” from experiment 1 were excluded from further analysis due to low cell counts and low number of genes per cells. Using Seruat^66^, the remaining experimental replicates were normalized, integrated, variable genes calculated (with vst), expression scaled, dimensionality reduced with principal compontent analysis (PCA), and clustered with Louvain clustering. The expression of cell cycle-associated genes, derived from^67^, was calculated with AddModuleScore and regressed out during scaling of the gene expression with linear regression. For clustering and dimensionality reduction, the number of principal components (PCs) were set to 13 based on ElbowPlot and DimHeatmap. Next, the data was analyzed to remove potential doublets, represented as cells clustering due to high or low gene and read counts, and contaminating cells, such as T-cells which were recognized based on the co-expression of DEGs specific for the cell type (*CD28, CD247, CD69, Ctla2a, Txk, Il21r* and *Rantes/Ccl5*). Seurat’s FindAllMarkers was used to compute differential gene expressions using a non-parametric Wilcoxon rank sum test. The remaining cells from each analytical group were integrated into a single data set. The pooled data set was dimensionality reduced with PCA, followed by UMAP, and re-clustered based on the same 13 PCs as in the previous step.

Trajectory inference was performed with tSPACE^36^. The trajectory PC (tPC) outputs were dimensionally reduced with UMAP to 2 and 3 dimensions. Clustering was performed with Louvain clustering for Seurat also using the tPCs as input. Pseudotime was averaged from the trajectories calculated by tSpace starting in cluster 1 (n = 9).

Differential gene expressions for cluster, infected versus bystanders and model comparisons were calculated with Seurats FindAllMarkers using non-parametric Wilcoxon rank sum test. The cut-off for the average log fold change was 0.25 for the cluster comparison, and 0.5 for the infected versus bystander respectively model comparison (p-value < 0.05 were used for all comparisons). The pseudobulk for heatmaps is based on cluster-averaging of gene expressions, calculated with Seurats AverageExpression function. The expression of genes upregulated in infected respectively bystander cells was calculated as a gene module with AddModuleScore.

The heterogeneity of identified clusters were analyzed using the Pathway RespOnsive GENes (PROGENy) package, which estimates the activity of 14 signaling pathways within cells using expression levels of specific gene sets^39–41^. The Gene Ontology Biological Process 2023 (Enrichr) tool were used to analyze pathways enriched within calculated differential gene expression data and visualized using the R library ggradar.

### Statistical analysis

Statistical analyses were performed using GraphPad Prism 9 software. One-way ANOVA with Tukey’s comparison tests was used to compare more than two groups, while two-way ANOVA with Tukey’s or Sidak’s comparison tests was employed for analyzing kinetic experiments. A significance level of p < 0.05 was considered significant, denoted by asterisks as follows: *p < 0.05, **p < 0.01, ***p < 0.001, ****p < 0.0001.

## Supplementary Information

Supplementary Figure S1.

Supplementary Table S1.

Supplementary Table S2.

## Data Availability

The single-cell RNA-sequencing data have been deposited in NCBI’s Gene Expression Omnibus (GEO) and are available under the GEO series accession number GSE235124.
