# Supplementary material for: In vivo regulation of the monocyte phenotype by Mycobacterium marinum and the ESX-1 type VII secretion system
Source: Sci Rep. 2025 Feb 7;15:4545. doi: 10.1038/s41598-025-88212-z (PMC11802795; doi:10.1038/s41598-025-88212-z)
Supplement: Supplementary file 1 — Supplementary Figure S1. [file 41598_2025_88212_MOESM1_ESM.pdf]

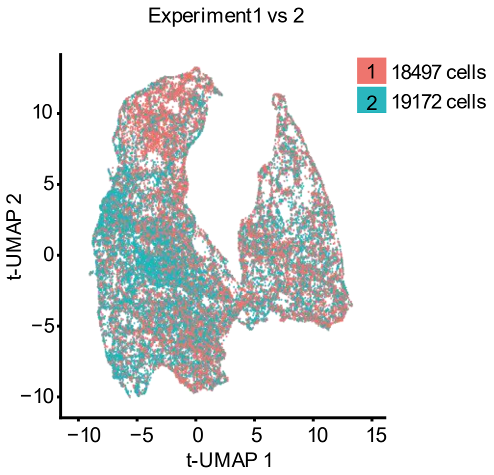

Supplementary Figure S1. Consistent representation of Ly6C$^+$MHCII$^+$CD64$^+$ myeloid cells in tUMAP across two independent experiments. (**A**) tUMAP visualization of scRNA-seq data obtained from infected and bystander Ly6C$^+$MHCII$^+$CD64$^+$ myeloid cells depicting the number and location of cells from two independent experiments**.**

Supplementary Table S1. Total DEGs for infected versus bystander cells. Average log fold change >0.5, p-value <0.05, consistent across the two experiments.

Supplementary Table S2. Total DEGs for WT versus ΔRD1 bystander cells. Average log fold change >0.5, p-value <0.05, consistent across the two experiments.
